# Nonlinear and threshold effects of built environment on older adults’ walking duration: do age and retirement status matter?

**DOI:** 10.3389/fpubh.2024.1418733

**Published:** 2024-06-28

**Authors:** Jiani Wu, Chaoyang Li, Li Zhu, Xiaofei Liu, Bozhezi Peng, Tao Wang, Shengqiang Yuan, Yi Zhang

**Affiliations:** ^1^State Key Laboratory of Ocean Engineering, Shanghai Jiao Tong University, Shanghai, China; ^2^JSTI Group, Nanjing, China; ^3^Key Laboratory of Advanced Public Transportation Science, China Academy of Transportation Sciences, Ministry of Transport, Beijing, China; ^4^Shanghai Municipal Engineering Design Institute (Group) Co., Ltd., Shanghai, China; ^5^Institute of Healthy Yangtze River Delta, Shanghai Jiao Tong University, Shanghai, China

**Keywords:** built environment, machine learning, older adults, age differences, retirement status, walking duration

## Abstract

**Introduction:**

Walking plays a crucial role in promoting physical activity among older adults. Understanding how the built environment influences older adults’ walking behavior is vital for promoting physical activity and healthy aging. Among voluminous literature investigating the environmental correlates of walking behaviors of older adults, few have focused on walking duration across different age groups and life stages, let alone examined the potential nonlinearities and thresholds of the built environment.

**Methods:**

This study employs travel diary from Zhongshan, China and the gradient boosting decision trees (GBDT) approach to disentangle the age and retirement status differences in the nonlinear and threshold effects of the built environment on older adults’ walking duration.

**Results:**

The results showed built environment attributes collectively contribute 57.37% for predicting older adults’ walking duration, with a higher predicting power for the old-old (70+ years) or the retired. The most influencing built environment attribute for the young-old (60–70 years) is bus stop density, whereas the relative importance of population density, bus stop density, and accessibility to green space or commercial facilities is close for the old-old. The retired tend to walk longer in denser-populated neighborhoods with better bus service, but the non-retired are more active in walking in mixed-developed environments with accessible commercial facilities. The thresholds of bus stop density to encourage walking among the young-old is 7.8 counts/km^2^, comparing to 6 counts/km^2^ among the old-old. Regarding the green space accessibility, the effective range for the non-retired (4 to 30%) is smaller than that of the retired (12 to 45%).

**Discussion:**

Overall, the findings provide nuanced and diverse interventions for creating walking-friendly neighborhoods to promote walking across different sub-groups of older adults.

## 1 Introduction

Population growth and population aging are major global demographic trends. According to the latest United Nations report (1), there were 771 million persons aged 65 years or above globally in 2022, with over one-quarter residing in Asia, North America, and Europe (2). Asia is anticipated to experience the most rapid growth in older populations from 2022 to 2050 (2). For example, being the most populous country in Eastern and Southeastern Asia, almost 35 percent of the Chinese population will be expected to age 65 and above by 2050 (3). These demographic trends attract attention to the health of older adults.

Participating in physical activity provides numerous health advantages for older individuals, lowering the risk of chronic diseases, bone fractures and disability (4, 5). For older adults, walking stands out as a form of low-intensity physical activity, characterized by its affordability, minimal risk, and seamless integration into daily routines (6, 7). The importance of walking extends beyond physical health to include social and psychological benefits, such as reducing feelings of loneliness and improving mood and cognitive function (8). Walking duration significantly influences the walking behaviors of older adults, playing a vital role in determining their weekly walking levels (9). Holding other factors constant, walking for more than 120 min per week is linked to improve cardiovascular health, enhance mental well-being, and better overall physical fitness (10). For older adults, participating in routine walking is essential for enhancing quality of life and promoting healthy aging (11).

Older adults exhibit a heightened susceptibility to the built environment owing to their proclivity for undertaking shorter journeys, their necessity for secure infrastructural facilities, and their dedication to a substantial duration of recreational activities in parks (12). There is growing studies evidence that a walk-friendly urban setting, featuring compact structures, connected pathways, accessible amenities, and green spaces, strongly influences older adults’ walking behaviors (13–15). Despite numerous efforts, our understanding of the links between the built environment and walking behaviors is still inadequate. First, the influence of a built environment variable on the duration of walking may exhibit variability across distinct intervals of the variable, with its efficacy potentially saturating at a specific threshold (16, 17). Previous studies do not adequately consider the large disparity in individual characteristics. Personal preferences, social attributes, and physical health lead to diverse travel behaviors. The built environment may exert disparate effects on distinct age cohorts (6). Moreover, changes in life stages often lead to adjustments in travel behavior (18). Second, many studies emphasized the linear influence of neighborhood environment on walking and neglected the irregular nonlinearity exhibited by neighborhood environment (19). Furthermore, there might be a point where a certain feature starts to significantly increase walking (trigger effect), and a point beyond which further increases in that feature do not make much difference (ceiling effect). (20). Consequently, the oversimplification of this association, exclusively within linear paradigms may engender bias in estimations (20, 21).

To fill these gaps, we employ the gradient boosting decision tree (GBDT) methodology on the dataset acquired from the travel survey conducted in the Zhongshan area. This study aims to address two primary research inquiries: (1) Whether the collective contribution of spatial attributes differs in associations between specific age groups and varies with different retirement statuses? and (2) What are the nonlinearities and thresholds of neighborhood environment in older adults’ walking duration? The answers to the inquiries will contribute to the strategic formulation and establishment of a more conducive built environment, fostering healthful living and enhanced mobility among the older demographic.

The subsequent sections of the manuscript are structured in the following way. Section 2 reviews the effects of existing literature, along with methods and findings related to studying relationships. Section 3 presents the research data, variables, and methodology. Section 4 explains the research findings. Section 5 provides concluding remarks. The last section shows the limitations.

## 2 Literature review

### 2.1 Neighborhood environment and older adults’ walking duration

Physical activity confers substantial health advantages upon older adults, mitigating the occurrence of chronic diseases, bone fractures, cognitive decline (22), enhancing mood and mental well-being, and reducing risk of falls in older adults (23). Older adults frequently engage in walking as a prevalent and preferred form of physical activity (9). Walking duration can increase overall physical activity, playing a significant role in promoting a healthy lifestyle (24). Therefore, exploring the neighborhood environmental correlates of walking duration in older adults is helpful to healthcare providers and urban planners.

Investigating the built environment’s effects on the walking behaviors of older adults has spurred the development of evidence-based interventions aimed at promoting walking activity in this population with a focus on health outcomes. Recent systematic reviews and meta-analysis have elucidated compelling evidence supporting beneficial connections between the following built environment features and walking (25–27): (1) walkability, the degree to which an area or neighborhood is conducive to pedestrian activities (26, 28); (2) access to/availability of services (29); (3) streetscape and pedestrian infrastructure, including sidewalks and walking trails, pedestrian-friendly features, sitting facilities, and streetlights (13, 30); (4) street connectivity (26); (5) safety and traffic, e.g., pedestrian safety, motorized traffic volume, and crime (31); and (6) esthetics, e.g., greenery and scenery (32, 33). Existing literature on transportation (or utilitarian) walking suggests that neighborhoods with high walkability, excellent street connectivity, and proximity to various destinations and services reported higher rates of transportation walking (34, 35). Although numerous empirical studies propose that enhancing built environment indicators can increase walking activity, less attention has been concentrated on modifying the built environment to enhance older adults’ walking behavior.

The associations between the built environment and walking behavior exhibit ambiguity and diversity (36, 37). Recent investigations have analyzed the nonlinear connections between walking behavior and the built environment (17, 20, 38). This nonlinear association is understandable because tend to derive enjoyment from walking when distances are not overly extensive; however, there may be a possible ‘trigger’ and ‘ceiling’ effects (20). Using data from Nanjing, China, and a random forest method, Cheng et al. revealed that built environment attributes affect walking time nonlinearly and matter only at certain levels (39). The findings indicated that the effective ranges of population density and land use mix stimulating walking are 6–20 persons/1000 m^2^ and 0.4–0.7 score. Beyond this ranges, they are related to a decline in walking. Nonlinearity suggests that an explanatory variable’s marginal effect on the outcome depends on that variable’s value (40). The influence of the variable may vary across distinct ranges, with its impact saturating upon reaching a specific threshold level (41).

### 2.2 Age and retirement status differences in the environmental correlates of travel behavior among older adults

Studies have demonstrated that built environment variables remarkably influence older adults’ travel behaviors (42). However, diverse travel behaviors among older adults are influenced by individual preferences, social factors, and physical health considerations (43). For example, some scholars found that older adults who live alone exhibit a decreased inclination for travel in comparison to their counterparts residing with their children (44, 45). Studies also suggested that older individuals in good health exhibit a greater propensity to opt for car travel (46). Furthermore, there is a notable reduction in travel time as individuals age, with a significant decline evident in those aged 75 and above (47). Therefore, it is necessary to analyze older adults’ travel behaviors in diverse aspects for effective segmentation.

Studies have found that healthcare expenditures for individuals aged ≥75 years (referred to as the old-old) are nearly double those of their younger counterparts. Additionally, the old-old exhibit heightened vulnerabilities in physical, mental, and financial domains in comparison to the young-old (48). Consequently, studying a broad age range of older adults (60–95 years) may obscure distinctions in travel behavior and health status between the young-old and old-old (49). Recent studies indicate that the old-old population demonstrates a slower walking pace, shorter steps, and increased step width in comparison to the young-old (50). Hence, there is a necessity to evaluate older adults’ walk behavior distinctly for the young-old and the old-old. Among previous studies on the built environment and walking behavior of older adults (31, 51), few have explored the subtle distinctions in characteristics between the young-old and the old-old age groups. Due to the scarcity of studies and conflicting findings on the diverse effects of the built environment on walking behavior in distinct age groups of older adults, additional research is warranted.

The travel behavior of older adults can vary based on factors such as retirement status and behavioral changes (48). Most older adults have more free time after retirement and prefer spending it outdoors (52). For example, in China, the legal retirement age is 50–55 for the female and 60 for the male. Notably, many individuals in China opt for re-employment after retirement (53). Examining the influence of retirement on walking provides valuable insights into the diverse travel patterns among older adults. However, such research is scarce.

## 3 Data and methods

### 3.1 Study area

**Figure 1:**
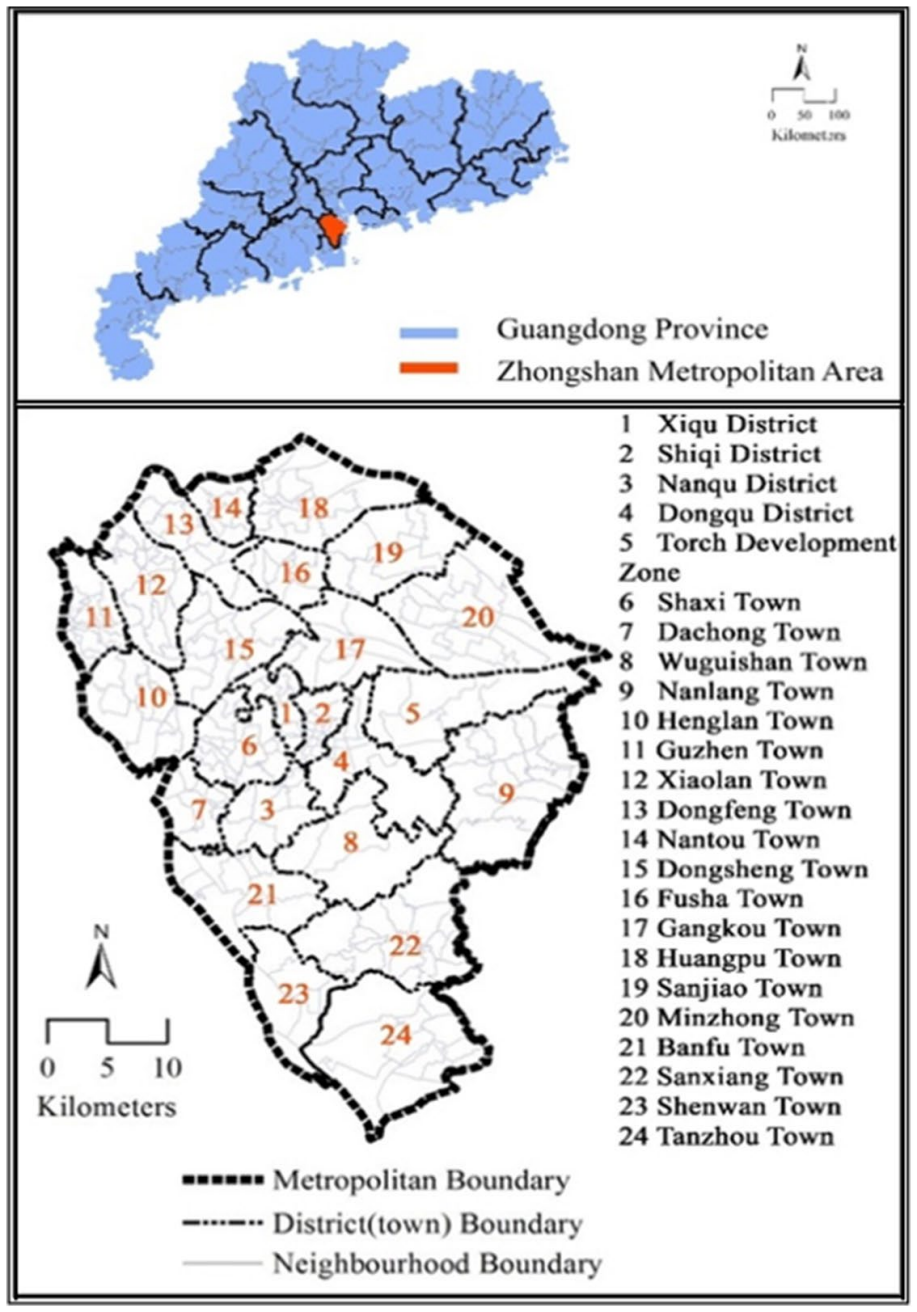
Study area.

This study focuses on Zhongshan City in Guangdong-Hong Kong-Macao Greater Bay Area (the Great Bay Area) of China to investigate the walking duration of older adults in the developing context (Figure 1). As a medium-sized city in the Great Bay Area, Zhongshan shares competitive economies and comparable levels of urbanization and motorization, and urban transport characteristics with similar cities in developing countries (11). Therefore, findings in Zhongshan are likely to be representative and informative for cities of this type. The average walking duration for older adults in Zhongshan is 18.84 min per day.

### 3.2 Data collection

This study employed the walking duration data from Zhongshan Household Travel Survey (ZHTS) in 2012. Adopting a stratified random sampling approach, the sample comprised 4,329 older adults, yielding a sampling rate of approximately 2%. In China, older adults aged 60–69 years are classified as young-old and 70+ years as old-old (54). In this study, the respondents consisted of 2,979 young-old and 1,350 old-old. The ZHTS 2012 facilitated the collection of self-reported data on walking activity, encompassing details on frequency, duration, and purpose of walking trips, in conjunction with socio-demographic information pertaining to respondents.

The dataset for characterizing built environment attributes encompasses: (1) delineations of neighborhood boundaries; (2) classifications of land use; (3) information on neighborhood population; (4) depictions of road networks; (5) locations of public transportation station; and (6) demarcations of political boundaries. All data were sourced from the Zhongshan Urban Planning Bureau and subsequently integrated into ArcGIS for further analytical exploration.

### 3.3 Main variables

This study categorized the main independent variables into personal-level socio-demographics, household-level socio-demographics, and the built environment. Personal-level socio-demographics include sex, age group (young-old and old-old), retirement status (retired or not), and attitude towards walking. Household-level socio-demographics includes household size (1, 2, and 2+ persons), income level (<20,000¥ CNY, 20000–60,000¥ CNY, and > 60,000¥ CNY per year), and numbers of bicycles, e-bikes, motorcycles, and private cars.

For the built environment attributes, we employed the widely-used “five Ds” developed by Ewing and Cervero (55). The “five Ds” refer to key dimensions of the built environment that influence walking. They are density, diversity, design, destination accessibility, and distance to transit. Additionally, we added Aesthetic as the sixth category of the built environment. To mitigate multicollinearity of independent variables, a Pearson correlation analysis was conducted to select one variable for each built environment category. See (38) for a detailed. The definitions and descriptions of both dependent and independent variables utilized in this study are outlined in Table 1.

**Table 1:** Variable description (sample size = 4,329).

Variables	Description	Type	Mean/ (%)	St.Dev.
Dependent variables				
Duration	Individual participant’s walking trip duration, minutes/day	Continuous	18.84	23.33
Personal socio-demographics				
Sex	Male	Male = 1	60.43	\
	Female	Female = 0	39.57	
Young-old	Average age of the young-old (60 to 69 years old)	Continuous	63.28	2.79
Old-old	Average age of the old-old (over 69 years old)	Continuous	75.39	4.93
Retired	The respondent is non-retired	Non-retired = 1	24.46	\
	The respondent is retired	Retired = 0	75.54	
Prowalk	The respondent prefers walking over other modes	Yes = 1	26.82	\
	The respondent prefers other modes over walking	No = 0	73.18	
Household socio-demographics				
HH-1	The household comprises a single individual	Yes = 1, No = 0	26.82	\
HH-2	The household comprises two individuals	Yes = 1, No = 0	35.34	\
HH > 2	The household comprises more than two individuals	Yes = 1, No = 0	37.84	\
Highinc	The household income is high (>60,000¥/yr.)	Yes = 1, No = 0	15.25	\
Midinc	The household income is medium (20000–60,000¥/yr.)	Yes = 1, No = 0	47.82	\
Lowinc	The household income is low (<20,000¥/yr.)	Yes = 1, No = 0	36.93	\
Bikes	The number of household bicycles	Continuous	0.61	0.71
E-bikes	The number of household e-bikes	Continuous	0.22	0.46
Motors	The number of household motorcycles	Continuous	0.76	0.85
Cars	The number of household private cars	Continuous	0.17	0.44
Built environment at the neighborhood level				
Popden	Population density (as Density), 1,000 persons/km^2^	Continuous	8.08	10.23
Mixture	Land use mixture (as Diversity). Entropy Index calculated on land uses	Continuous	0.70	0.18
Sidewalk	Sidewalk density (as Design). Length of sidewalk per km^2^, km/ km^2^	Continuous	4.65	3.31
Bus stop	Bus stop density (as Distance to transit). Number of bus stops counts per km^2^	Continuous	1.78	2.50
Comacc	Commercial accessibility (as Destination accessibility). Commercial establishments’ spatial extent within a 1 km radius from the neighborhood center, in ha	Continuous	33.19	33.08
Green	Green space accessibility (as Aesthetic). Proportion of green space in the total land use,	Continuous	0.07	0.08

### 3.4 Model specification

This research applied the GBDT method, a recently developed approach originating from computer science (56). This method is increasingly used to analyze the relationship between the built environment and travel (36, 57). Gradient Boosting is a major category of algorithms within the Boosting framework. The fundamental concept is to train new weak classifiers based on the negative gradient information of the current model’s loss function, and then integrate these well-trained weak classifiers into the existing model in an accumulative manner (58). The basic process of the algorithm is as follows: in each iteration, first calculate the negative gradient of the current model on all samples, then train a new weak classifier with this value as the target for fitting and calculate the weight of this weak classifier, ultimately achieving an update to the model. GBDT integrates decision tree and gradient boosting approaches, minimizing a loss function to approximate the actual value (56). Each tree in the ensemble learns the residual (difference) of the sum of all the preceding tree predictions, allowing GBDT to handle irregular nonlinear relationships effectively. This residual represents the cumulative amount needed to reach the true value after adding the predicted value.

GBDT offers several advantages for this study. First, it adeptly addresses irregular nonlinear relationships compared to conventional models. Second, it predicts the significance of independent variables without predetermined linear assumptions, facilitating the comparison of their roles. Third, the analysis includes creating partial dependence plots (PDPs) to depict the connections, considering interactions with other independent variables (59). These advantages assist in determining potential threshold effects and effective impact ranges of land-use policies on older adults’ walking duration. However, GBDT has limitations, including the inability to calculate *p*-values for statistical inference and susceptibility to overfitting (17).

**Figure 2:**
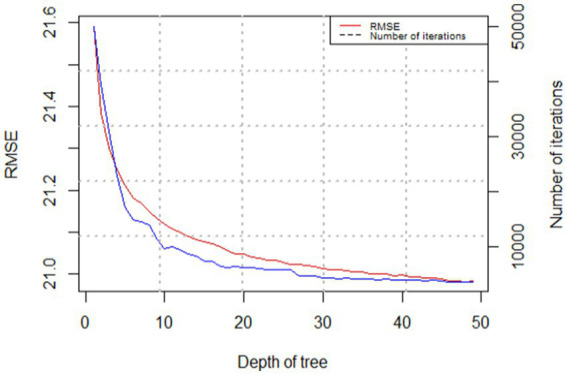
RMSE and number of iterations versus tree depth.

**Figure 3:**
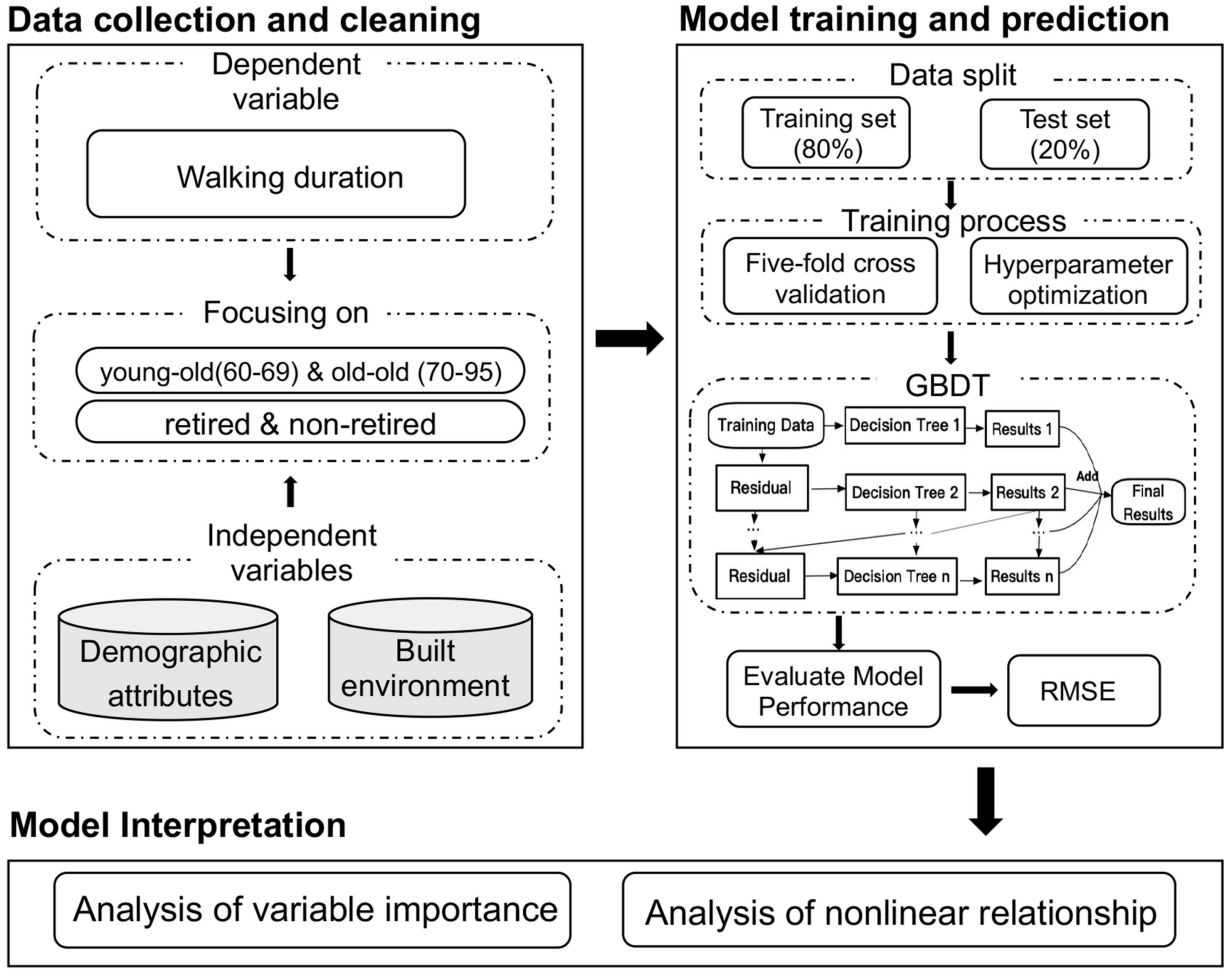
Analysis framework.

We applied the gbm package (60) in R to estimate GBDT models. When it comes to estimating models, three key factors make all the difference: tree depth, learning rate, and number of trees (58). Tree depth is how many layers a decision tree has, which shows how complex the tree is. Learning rate how much to weigh each tree’s guess when we are building our final model. It ranges from 0 to 1. Number of trees is how many trees are in our forest working together. As the depth of the tree increases, both RMSE and the number of iterations decrease (Figure 2). We set tree depth to be 45 and learning rate to be 0.001, and used five-fold cross validation to search for the optimal number of trees by which the model generates the smallest root mean squared error. Cross-validation is a statistical method used to estimate the skill of machine learning models. The five-fold cross-validation is a specific type of cross-validation, which can maximize the use of the available data. This approach ensures robustness and reliability in the evaluation of the model. Final Model has 3,670 trees, respectively. The analysis process is summarized in Figure 3.

## 4 Results and discussion

### 4.1 Relative importance (RI)

#### 4.1.1 The RI of independent variables

The relative importance of independent variables in predicting the relationship between the built environment and walking duration among older adults as a percentage is shown in Table 2. All these variables collectively contribute to 100% relative importance. Specifically, built environment variables account for 57.37% of the total variances among independent variables. Personal and household variables contribute 23.75 and 18.88%, respectively. This outcome emphasizes the crucial role played by the built environment in influencing the walking behaviors of older adults. It adds further support to recent studies highlighting the substantial influence of built environment variables on travel outcomes (15, 31).

**Table 2:** Relative importance of personal, household, and built environment variables.

Parametric variables	Ranking	Relative importance (%)	Sum (%)
Personal variables			23.75%
Gender	10	3.76	
Age	1	15.58	
Retired	11	2.58	
Prowalk	15	1.83	
Household variables			18.88%
HH-1	17	1.06	
HH-2	14	1.97	
Highinc	16	1.12	
Midinc	12	2.26	
Bikes	8	5.11	
E-Bikes	13	2.10	
Motors	9	4.37	
Cars	18	0.89	
Built environment variables			57.37%
Popden (Population density)	3	11.39	
Mixture (Land use mixture)	4	8.75	
Sidewalk (Sidewalk density)	7	7.18	
Bus stop (Bus stop density)	2	13.98	
Comacc (Commercial accessibility)	6	7.92	
Green (Green space)	5	8.15	

Regarding the built environment variable, bus stop density has the most considerable contribution (RI = 13.98%) in predicting older adults’ walking duration. This finding is rational considering the low prevalence of older drivers in China (3.7%) compared to Western countries (61). Due to this low prevalence of older drivers, older adults in China rely more heavily on public transportation to meet their mobility needs. Public transportation is the primary mobility option for Chinese older adults, especially in medium and long distance trips (42). A higher density of bus stops means that older adults have better access to public transportation options, which can significantly influence their walking behavior. As suggested in recent studies, walking serves as the essential mode in the first/last mile to transit service (62). Population density is another significant variable with RI over 10% (11.39%). The results demonstrate the importance of dense development and convenient transit service in encouraging longer walking among older adults.

Notably, age emerges as the most crucial predictor of walking duration among all independent variables (RI = 15.58%). Previous research indicated that older adults’ travel time decreases with age (47). Therefore, this study delves further into age differences in the nonlinearities of the built environment. Additionally, retirement status, influencing older adults’ psychological aspects such as lifestyle and habits, is also examined for heterogeneity in the nonlinearities of the built environment.

#### 4.1.2 The age and retirement status differences in RI

Table 3 includes the RI of independent variables for older adults with different age and retirement status. For the old-old and non-retired, the accumulated contributions of built environment attributes are larger than their young and retired counterparts. This aligns with prior research (63) that older or non-retired Individuals generally place greater importance on perceptions of the built environment in travel-related decision-making.

**Table 3:** Relative importance of age group and retirement status.

	Age group				Retirement status			
Parametric Variables	Young-old (60-69)		Old-old (70+)		Retired		Non-retired	
	Ranking	RI (%)	Ranking	RI (%)	Ranking	RI (%)	Ranking	RI (%)
Personal	Sum of RI: 36.46		Sum of RI: 25.84		Sum of RI: 21.63		Sum of RI: 22.22	
Gender	12	8.43	10	3.79	10	3.55	8	5.98
Age	7	6.90	1	11.85	1	16.50	1	13.08
Retired	11	3.05	15	1.38	–	–	–	–
Prowalk	1	18.08	6	8.82	14	1.58	12	3.16
Household	Sum of RI: 14.83		Sum of RI: 18.28		Sum of RI: 19.70		Sum of RI: 18.36	
HH.1	17	0.82	14	1.73	17	0.97	13	2.53
HH.2	13	2.08	13	2.15	11	2.29	15	1.20
Highinc	16	1.75	17	1.20	15	1.20	17	0.41
Midinc	15	1.67	12	2.38	12	2.22	11	3.44
Bikes	10	3.11	9	5.17	8	5.28	9	5.05
E-bikes	14	1.68	16	1.30	13	2.19	14	1.43
Motors	9	3.11	11	3.58	9	4.48	10	3.84
Cars	18	0.61	18	0.77	16	1.07	16	0.46
Built environment	Sum of RI: 54.59		Sum of RI: 55.88		Sum of RI: 58.67		Sum of RI: 59.42	
Popden	3	8.49	2	11.03	3	11.08	4	10.32
Mixture	8	6.78	7	7.79	4	8.45	2	10.81
Sidewalk	6	7.38	8	7.35	7	7.28	6	8.99
Bus stop	2	15.47	3	10.51	2	15.30	7	8.68
Comacc	5	8.04	4	9.67	6	8.20	3	10.41
Green	4	8.43	5	9.53	5	8.36	5	10.21

For both age groups, population density and bus stop density emerge as the two most crucial built environment variables. Bus stop density has the largest contribution (15.47%) among the young-old, while the second largest among the old-old (10.51%). This aligns with the expectation that young-old, being relatively healthy and active, may travel more frequently with transit and generate longer walking for the first/last mile. On the contrary, population density exhibits the most significant role (11.03%) among the old-old and the second most (8.49%) among the young-old. The result indicates that higher population density might signify less safe walking for the old-old, given their potential mobility and health limitations. High population density can lead to increased traffic congestion, higher pedestrian-vehicle conflicts, and crowded sidewalks, which pose significant challenges and safety risks for older adults with reduced mobility and health issues. Research has shown that higher traffic volumes and crowded conditions have been associated with an increased risk of falls and accidents among older adults (64). Moreover, the complexity of navigating densely populated areas can discourage walking and negatively impact the perceived and actual safety of older pedestrians (65).

For the two groups with different retirement status, the performance of built environment variables is diverse. First, bus stop density has the largest contribution among the retired (15.30%), but a much smaller contribution among the non-retired (8.68%). This finding aligns with previous research indicating the retired prefer public transport (66). Second, land use mixture (10.81%) is the most critical built environment variable for the non-retired, while ranking only fourth for the retired. This may because mixed land use is more convenient for commuting by walking for the non-retired older adults. Third, commercial accessibility ranks third for the non-retired but sixth for the retired. This result aligns with a previous study indicating a significant increase in joint distance for diverse out-of-home activities in retirement (67) and suggests that commercial establishments within a 1 km radius from the neighborhood center are more appealing to non-retired older adults.

### 4.2 Relationships between walking duration and built environment variables

#### 4.2.1 Nonlinear and threshold effects on older adults’ walking duration

**Figure 4:**
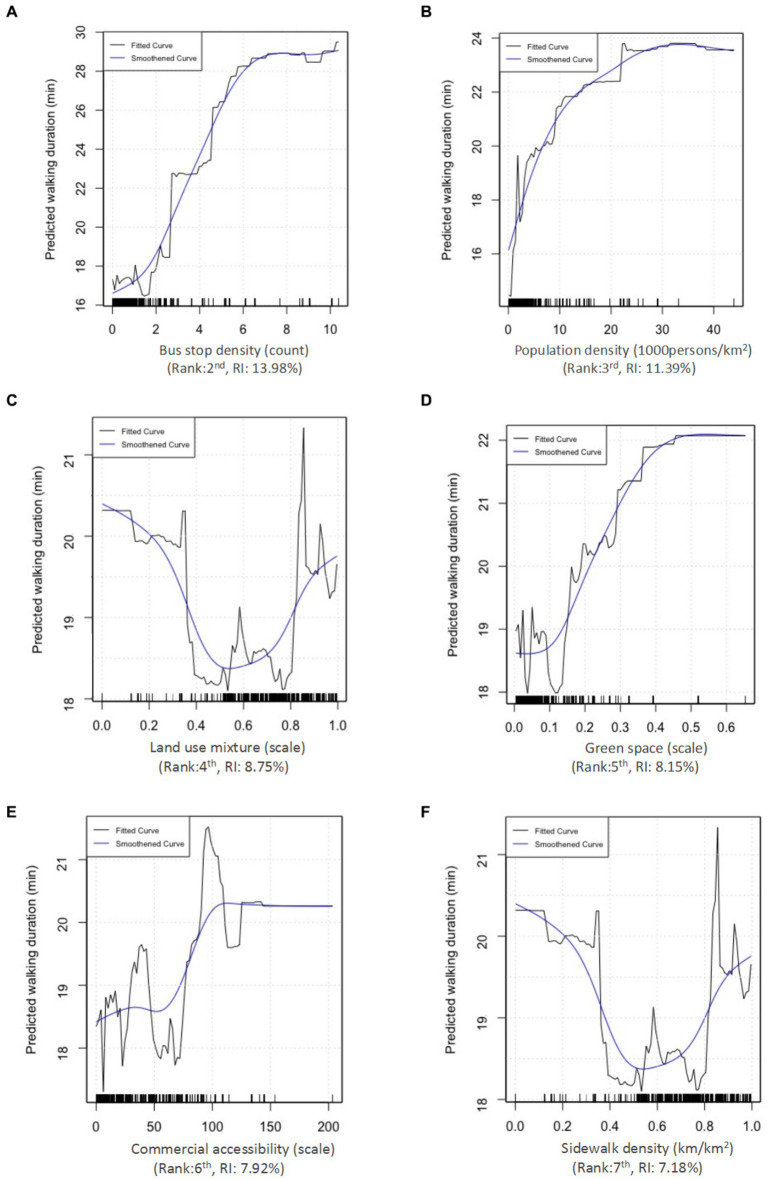
Nonlinear effects of the built environment on the walking duration of older adults include various factors: **(A)** Bus stop density, **(B)** population density, **(C)** land use mixture, **(D)** green space, **(E)** commercial accessibility, and **(F)** sidewalk density.

We use PDPs to depict the connections between built environment attributes and the predicted walking duration (Figure 4). The vertical axes of these PDPs represent the predicted marginal effect. In addition to the fitted curves, we apply smoothing techniques to accentuate the overall trends in the relationships. The subsequent discussion outlines the results in order of their relative importance. Overall, the six variables show nonlinear threshold effects, albeit in different degrees. These findings are essential for planners and policymakers to achieve effective interventions.

As shown in Figure 4A, there exists a positive correlation between walking duration and bus stop density. The walking duration increases from 16 to 28 min when bus stop density rises from 0 to 7 counts/ km^2^, and it stabilizes beyond 7 counts/ km^2^. This indicates walking duration increased with bus stop density up to a point (7 counts/km^2^), but beyond this point, further increases in bus stop density did not lead to significant changes in walking duration. As suggested by previous literature, walking is an essential mode for the first/last mile of public transport (62), thus an increased density of bus stops indicates a higher volume of pedestrian activity. This interpretation supports the identified threshold effect of bus stop density on walking duration among older adults. Therefore, prioritizing a bus stop density of 7 counts/ km^2^ is recommended to enhance walking among older adults in Zhongshan.

Similarly, population density is positively related to walking duration beyond 30,000 people/km^2^ (Figure 4B), in line with existing literature (68). Once the population density surpasses 30,000 people/km^2^, walking duration becomes stable at around 23 min/day. This suggests that an optimal promotion of walking among older adults may be achieved through a moderate-to-high density of urban developments. It is reasonable that dense environments support older adults’ walking by providing access to diverse destinations via well-connected street networks (68). Additionally, densely populated neighborhoods typically boast a greater presence of walking infrastructure and public facilities (69). However, the nonlinear effects become marginal and slightly adverse when population density exceeds 35,000 people/km^2^. This is likely due to the increased risk of injury in highly dense areas (39). However, caution is warranted in interpreting this negative correlation due to the sparse distribution of the data.

Regarding the land use mixture, the nonlinear relationship shows a U-shaped curve with walking duration (Figure 4C). When the land use mixture is less than 0.6, it has a negative effect. Beyond the threshold of 0.6, the walking duration substantially increases, with the most effective and reliable range identified between 0.81 and 0.85. This finding is consistent with our intuition and in line with previous studies that mixed development improves walkability for older adults by increasing the possibility of trips of short to moderate distances instead of long-distance ones, which allows easy access to different types of destinations (70–72).

As shown in Figure 4D, green space positively correlates with walking duration among older adults and the threshold is around 0.45. Walking duration remains nearly constant when the ratio of land used for green spaces is below 0.1. However, the walking duration upsurges by about 3.4 min when the ratio of land used for green spaces rises from 0.1 to 0.45, and the largest effect occurs when the green space reaches 0.47. The results indicate that the ratio of land used for green spaces is most effective within the range of 10 to 47%. Within this range, the relationship appears to be positively linear. This result aligns with some previous studies (20, 33, 73), suggesting that proximate or appealing green spaces exert a more substantial influence on fostering outdoor walking and increasing the walking duration of older adults.

Commercial accessibility positively correlates with walking duration (Figure 4E). The increase of walking duration is slight (at about 0.2 min) when commercial accessibility grows from 0 to 50. Then, a considerable increase (around 1 min) occurs when the number of bus stops falls within the range of 50 to 100. Finally, the effect of commercial accessibility becomes trivial when it exceeds 100. It is noteworthy that when commercial accessibility surpasses 110, the associated interval exhibits a limited number of sample points, thereby rendering the relationship within that interval less reliable for interpretation. This result suggests that improving the commercial accessibility of neighborhoods emerges as an approach to promote walking among older adults. The finding echoes the results of prior studies that high commercial accessibility provides facilities and services within short distances, particularly attractive to older pedestrians (31, 74).

As shown in Figure 4F, the sidewalk density has a V-shaped nonlinear association with older adults’ walking duration, and pivotal turning is at 6 km/ km^2^. Within the range of 0 to 6 km/km^2^, sidewalk density has a negative impact on walking duration. Subsequently, an increase in sidewalk density from 6 to 12 km/km^2^ corresponds to a rise in walking duration from 18.3 to 20.3 min. This finding is coherent, as higher sidewalk density implies a greater variety of walking routes, contributing to a pedestrian-friendly environment characterized by well-connected streets. This, in turn, encourages both utilitarian and recreational walking (75–77). This result is also consistent with the national design guidelines that recommends 6–10 km/km^2^ as the baseline sidewalk density (78).

#### 4.2.2 Nonlinear and threshold effect in different age and retirement status

**Figure 5:**
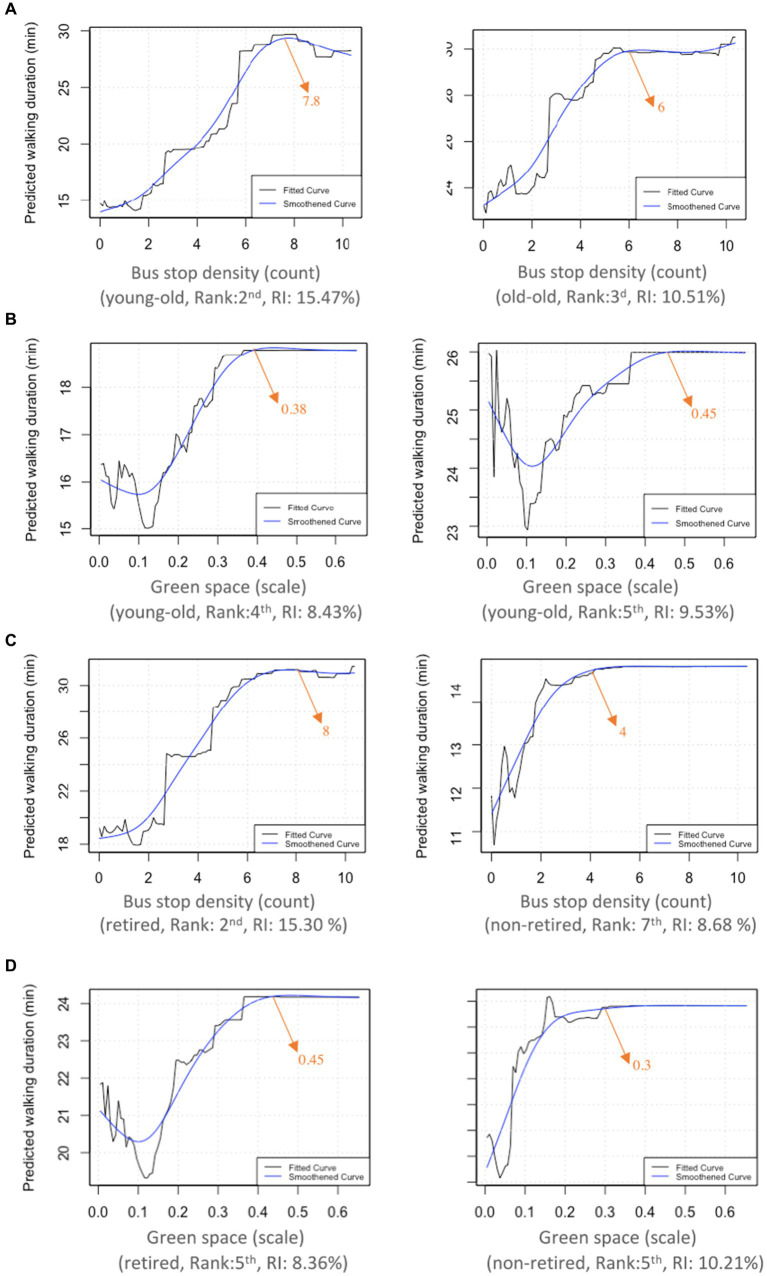
Bus top density differences between young-old and old-old **(A)**, green space differences between young-old and old-old **(B)**, bus top density differences between retired and non-retired **(C)**, and green space differences between retired and non-retired **(D)**.

Figure 5A shows the impact of bus stop density on the walking duration of the young-old and old-old. For the young-old, approximately 8 stops/km^2^ of bus stops is sufficient to optimize walking duration, whereas 6 stops/km^2^ of bus stops are most effective for the old-old. This means that the young-old might require a higher density of bus stops to achieve the same level of convenience and accessibility for walking as the old-old. This difference can be attributed to the reason that young-old, possessing more available time, may travel to more distant environments via public transit, while the old-old, facing worsening physical conditions, prefer to travel closer to home.

Figure 5B displays the effects of the ratio of land used for green spaces on the walking duration of the young-old and old-old. Positive threshold effects range from 12 to 38% for the young-old and 12 to 45% for the old-old. Correspondingly, the walking duration changes from 23 to 26 min for the old-old and 15 to 18 min for the young-old. These results suggest that the walking duration of the old-old are more likely to be influenced by the green space accessibility, possibly due to the decline in physical functioning.

Figure 5C reveal that, for the retired, approximately 8 stops/ km^2^ of bus stops are adequate to optimize walking duration, whereas 4 stops/ km^2^ are most effective for the non-retired. The scale of the influence of bus stop density on walking duration in the non-retired is smaller than that in the retired. The walking duration rises from 18 to 31 min for the retired when the number of bus stops increases from 0 to 8 per km^2^. However, the relevant magnitude in the non-retired is 4.6 min, ranging from 10.2 to 14.8 min. These findings may be attributed to the surplus time available to the retired older adults, enabling them to travel to more distant environments via transit. Regarding the percentage of green space (Figure 5D), the positive threshold effects range from 0.1 to 0.45 for the retired, surpassing those for the non-retired (0.04 to 0.3). Additionally, the retired exhibit longer walking durations within the threshold intervals. These results suggest that the retired are more sensitive to green space accessibility, possibly due to the fact that the retired have more time to visit green spaces such as parks and gardens.

## 5 Conclusions and policy implications

For the first time, this study explores the heterogeneity in the nonlinearities and threshold effects of the built environment among cohorts with differences in age and retirement status. The results contribute threefold to existing literature and planning practice on the built environment interventions for promoting walking.

Firstly, the findings indicate the prevalence of nonlinearities of the built environment on walking duration among older adults, aligning with recent literature (17, 20, 38). This challenges the conventional assumption of linearity in active travel studies and helps in better understanding the real relationships.

Secondly, this study assessed the significance of built environment characteristics in predicting the walking duration of older adults. These findings provide observational evidence of the varied impacts of the built environment on older adults’ walking behavior across distinct age groups and retirement statuses. Understanding the diverse impacts of built environment characteristics allow policymakers and planners to formulate customized policies to encourage walking among older individuals.

Thirdly, the results demonstrate the explicit thresholds of effective built environment interventions on walking duration of older adults. For instance, in Zhongshan, the 6 stops/km^2^ of bus stops are sufficient to optimize walking duration, and the positive threshold effects of green space coverage range from 10 to 45%. Furthermore, these two built environment characteristics show distinctive threshold effects with walking durations in groups with different age and retirement status. The observed threshold effects offer discriminating observations for policymakers and planners to create a more diverse and age-friendly environment for active travel.

In conclusion, our study provides new insights regarding the impacts of the built environment on older adults’ walking behavior and facilitates urban design and transportation planning initiatives on walking-friendly community.

## 6 Limitations

This study has several limitations. Firstly, the utilization of cross-sectional data in this study implies that the relationships are more correlational than causal. Secondly, this study did not consider the residential self-selection, a phenomenon where individuals select their living location in accordance with preferences (79). Subsequent research should incorporate more comprehensive indicators, such as respondents’ housing preferences, to explain the self-selection effect. Finally, the identified thresholds in this study might not be readily transferable to cities with differing characteristics in the built environment. Therefore, transferability requires future research.

## Data availability statement

The original contributions presented in the study are included in the article/supplementary material, further inquiries can be directed to the corresponding author.

## Author contributions

JW: Writing – review & editing, Writing – original draft, Methodology. CL: Writing – review & editing, Conceptualization. LZ: Writing – review & editing, Resources. XL: Writing – review & editing, Formal analysis. BP: Writing – review & editing, Methodology. TW: Writing – review & editing, Software. SY: Writing – review & editing, Project administration. YZ: Writing – review & editing, Supervision.

## Ethics statement

Ethical review and approval were not required for the study on human participants in accordance with the local legislation and institutional requirements. The participants provided their written informed consent to participate in this study.
